# Effects of extended reality on language learning: A meta-analysis

**DOI:** 10.3389/fpsyg.2022.1016519

**Published:** 2022-09-20

**Authors:** Jingying Chen, Jian Dai, Keke Zhu, Liujie Xu

**Affiliations:** ^1^School of Marxism, Zhejiang University of Technology, Hangzhou, China; ^2^School of Management, Zhejiang University of Technology, Hangzhou, China; ^3^College of Educational Science and Technology, Zhejiang University of Technology, Hangzhou, China; ^4^College of Foreign Languages, Zhejiang University of Technology, Hangzhou, China

**Keywords:** extended reality, virtual reality, augmented reality, language learning, meta-analysis

## Abstract

In recent years, there has been increasing use of extended reality (XR) in language learning. Many scholars have conducted empirical research on the relationship between the two, but conclusions have been inconsistent, which calls for an organization and reanalysis of relevant literature. Articles published between 2000 and 2022 on the impact of XR on language learning were retrieved from the Web of Science and Scopus databases, and 17 of them (including 21 independent samples and 993 subjects) were included in this meta-analysis. The findings indicate that XR could promote language learning (effect size = 0.825). The moderating effects of education level, target language, and technology type were also tested, and the results indicate that the target language type significantly moderated the effect of XR technology on language learning (*Q* = 30.563, *p* < 0.001). Moreover, based on the subgroup analysis, several research questions worthy of further exploration in this field are discussed. Some suggestions are provided, noting that these technologies should be personally designed for learners and learning objects when applied in order to improve the effects of language learning.

## Introduction

In the context of economic globalization and cultural diversity, language learning is an inevitable subject for all learners; therefore, it has become a popular research topic among scholars. Today, foreign language learning is still considered extremely difficult by a great number of learners. In response, schools have been trying to apply various technologies to foreign language education. Extended reality (XR), which gradually develops and matures, has garnered increasing attention (Chen, 2016; Luo et al., 2021b). This is because XR provides learners with the authentic language environment that language learning requires, thus enabling users to have a real learning experience (Moeller and Catalano, 2015).

XR is an umbrella term encapsulating augmented reality (AR), virtual reality (VR), and everything in between. AR and VR are two related but distinct technologies. VR, as the name suggests, is an immersive technology that simulates reality and provides users with real experiences (Radianti et al., 2020; Luo et al., 2021a), while AR is a technology that subtly integrates virtual information with the real world; that is, through extensive use of various technical means, the virtual information generated by computer, such as text, image, three-dimensional model, music, video, and so on, is simulated and applied to the real world, and these two types of information complement each other to “augment” the real world (Han et al., 2020; Lee, 2020). In contrast to VR, AR technologies are more effective at providing contextualized and socialized learning experiences (Lin et al., 2019). In the field of educational technology research, these technologies have been examined for a long time. The 2017 Horizon Report listed VR as the technology that holds great potential for boosting the development of education (Freeman et al., 2017). Moreover, in the 2020 EDUCAUSE Horizon Report, it was emphasized that the number of XR users had grown in recent years due to the decreasing cost and innovations in technical capabilities and immersive experience; XR had become an important technology to be implemented in the field of educational technology (Brown et al., 2020; Feng et al., 2021). Scores of scholars have noticed the potential applications of XR-related technologies, and there has been growing recognition among them that technological advances could decrease the costs of equipment and contribute to the promotion of technology (Hwang and Hu, 2013; Vapenstad and Buzink, 2013; Parong and Mayer, 2018; Li et al., 2021). Lee (2020) pointed out that in the past two decades, language learning and XR-related technologies have been very closely linked. Therefore, scholars have also begun to pay attention to this emerging topic. Furthermore, studies have verified that XR technology intervention is beneficial to language learning (Holden and Sykes, 2011; Liu et al., 2016; Binhomran and Altalhab, 2021; Nicolaidou et al., 2021; Zhang et al., 2022). The above findings provide the prerequisite for the educational transformation in language learning enabled by technology. At the same time, education researchers are obliged to explain changes in the education mechanism as well as improvement in learning performance that occurred after the integration of nascent technologies into education. By searching authoritative databases, including the Web of Science core collection and Scopus, we found no systematic meta-analysis of XR technology in language learning. Therefore, from the perspective of technological transformation, we adopted a method of comprehensive research (meta-analysis) that is scientific and systematic to evaluate XR’s impact on language learning. This paper reached convincing conclusions by synthesizing various empirical research studies. We mainly explored the following questions:

1. Does XR technology have a significant positive impact on language learning, and if so, what is the effect size?
2. Do education level, target language, technology type, and specific language skills moderate the effect of XR technology on language learning?
3. Based on systematic review and quantitative analysis, what are the potential research questions or research directions that need to be further explored in terms of the effects of XR technology on language learning?

## Literature review

XR has been integrated into language learning for a long time. Solak and Erdem (2015) traced research of this kind back to 1995, when they were conducting a content analysis of VR-assisted foreign language learning. However, a careful analysis of some research reveals that what they thought of XR at that time was very different from how it is understood today. For example, the understanding of virtual devices’ applications in learning was comparatively simple in the past, so the use of mobile headset virtual devices was considered an application mode of virtual devices (Tai and Chen, 2021). However, with updated technology, many devices have now become obsolete or are no longer classified as XR.

The integration of XR into language learning has another characteristic: empirical research is mainstream now, and many studies are experimental or quasi-experimental, which constitutes an important basis for the present paper. Experimental or quasi-experimental studies are often characterized by highly standardized procedures, strict control of irrelevant variables, high reliability and validity of measurement tools, and high repeatability of research conclusions. Huang et al. (2020) is an example of typical research on this topic. The team developed a Chinese writing learning system that was based on spherical video-based virtual reality (SVVR), and they carried out experiments in a senior high school writing class. They found that compared with the control group, whose learning was supported by conventional technology, the experimental group that used the SVVR system had better writing performance and self-efficacy. The team took a further step to measure the cognitive loads of the two groups, discovering that technology-supported learning can considerably reduce the cognitive loads of learners and boost their learning performance; Nicolaidou et al. (2021) also applied normative randomized control-group pretest-posttest design, indicating that VR applications can bring better immersion and engagement to learners through between-group comparisons (*t*-test). Moreover, it was the advantages of the application of technology in language learning that attracted the most attention of the researchers, and studies have revealed that the advantages include enhancing learners’ learning motivation, changing their learning attitude, and improving their academic performance (Wehner et al., 2011; Qiu et al., 2021; Tai and Chen, 2021).

Another prominent characteristic of the application of XR in language learning is its rapid growth in recent decades, which can be understood through a comparison of the following two studies. When Solak and Erdem (2015) were reviewing this research topic, they discovered that from 1995 to 2015, only 40 papers were published on applying XR in foreign language learning and teaching. However, Qiu et al. (2021) found that there had been up to 150 relevant studies published between 2008 and 2019. A systematic review of the literature revealed that the application of XR was mainly conducted in higher education. This seems easy to explain because colleges and universities have easier access to these technologies, and researchers are often university faculty, which facilitates their study of the topic.

Other scholars have conducted meta-analyses in studies relating to this topic. For example, Wang et al. (2020) reviewed and examined relative research on Three-Dimensional Virtual Worlds (3DVWs, a 3D game) in language learning between 2008 and 2019, including 13 articles, and found that the application of 3DVWs dramatically improved students’ attitudes and self-efficacy. Although 3DVWs are not exactly the same as the VR technology explored in this study (3DVWs are not considered VR technology without the use of a wearable VR device), Wang et al. (2020) was the first meta-analysis conducted on emerging technologies intervening in language learning and published in two authoritative indexes of the Web of Science core collection; therefore, it is of great significance for this study. Furthermore, it changes the situation where the only qualitative method was used to describe and summarize the study of XR applications in language learning, and it is another example of a successful meta-analysis conducted on a cutting-edge topic. Based on the above review, we find that there have been a large number of empirical studies on the use of XR in the field of language learning—enough to support a complete and systematic meta-analysis. Although some scholars have also conducted a meta-analysis (Wang et al., 2020), previous studies have not included both VR and AR in the investigation. Meanwhile, we are also concerned that a series of experimental studies closely related to the use of XR technology in language learning emerged from 2020 to 2022 (Chen and Hwang, 2020; Huang et al., 2020; Lee, 2020; Binhomran and Altalhab, 2021; Chen et al., 2021; Lai and Chen, 2021; Nicolaidou et al., 2021; Tai and Chen, 2021; Xie et al., 2021; Tai et al., 2022); therefore, it is necessary to implement a complete and systematic meta-analysis that includes this most recent work. The present study analyzed the research regarding the effectiveness of XR-assisted language learning in a quantitative manner, and confirmed the moderator variables that affect the validity of XR to better guide its future application in language learning.

## Materials and methods

### Definition and application of meta-analysis

The present study used a meta-analysis to analyze the effect of XR technology on language learning. Meta-analysis belongs to a branch of evidence-based research, and is capable of enhancing credibility by increasing sample size to resolve the inconsistencies of research results (Glass, 1976; Oswald and Plonsky, 2010). As a special method of systematic review, meta-analysis has the following three advantages (Lipsey and Wilson, 2001; Cooper, 2015):

1. Meta-analysis is a systematic review of the existing research, and is able to shed deep insights into the hypothesis, process, and conclusion of the included research.
2. Meta-analysis takes account of the strength of the effect in every case of empirical research, which can better satisfy the requirement for data, therefore offering a more convincing conclusion.
3. Meta-analysis employs a programmed method to deal with the information in a vast literature, enabling it to consider both the depth and the range of the studies’ contents.

Meta-analysis was first applied in clinical medicine and social psychology (Smith and Glass, 1977; Rosenthal and Rubin, 1978), and was then introduced in the field of education (Glass and Smith, 1979). Today, meta-analysis is widely applied to language learning. For example, Lin (2014) used it to study the impact of computer-mediated communication (CMC) on language learning; Plonsky and Ziegler (2016) used second-order analysis to explore the relationship between computer-assisted language learning (CALL) and second language acquisition (SLA). These successful cases demonstrated the application value of meta-analysis in education as well as in language learning. Thus, in this study, we sought to further develop educational technology by employing a meta-analysis to identify the impact of XR on language learning outcomes.

The software we used to carry out the meta-analysis was Comprehensive Meta-Analysis (CMA, version 3.0, developed by Wilson et al. in the United States and United Kingdom). To ensure that the research is scientific and precise, the present study followed the Preferred Reporting Items for Systematic Reviews and Meta-Analyses (PRISMA) guidelines (Moher et al., 2009), which include a literature search, literature screening, and data coding.

### Search strategy

According to the objective of this study, our team retrieved articles on the impact of XR on language learning outcomes from the Web of Science core collection (to ensure the research quality, only SCIE and SSCI indexes were selected from the Web of Science database) and Scopus databases between 2000 and 2022. After reviewing several relevant studies (Luo et al., 2021a; Villena-Taranilla et al., 2022) and several attempts, the final retrieval strategy was determined as follows:

TS = ((“VR” OR “virtual reality” OR “AR” OR “augmented reality” OR “MR” OR “mixed reality” OR “XR” OR “extended reality”) AND (“language learning”)).

As some articles were duplicated in the two databases, our research team deleted them when incorporating the data. Ultimately, 470 articles were procured before proceeding to the next stage.

### Inclusion criteria and assessment of quality

Meta-analysis should follow strict criteria when screening the included literature (Moher et al., 2009; Xu et al., 2022). First, the articles must involve quantitative empirical research, and literature that does not meet the requirements, such as review research, narrative studies, and qualitative research, should be excluded. Second, the research topic needs to be closely related to both XR technology and language learning. Third, the research method should be a rigorous and scientific experimental method. Finally, studies need to report the statistics necessary to calculate the overall effect size. The details of these research criteria can be found in Table 1.

**Table 1:** Inclusion criteria for articles.

Category	Inclusion criteria
Literature type	Quantitative empirical research; literature that does not meet the requirements, such as review research, narrative studies, and qualitative research, should be excluded
Research topic	The effects of XR, such as VR, AR, MR, etc., on language learning
Research method	The experimental method is the only approach, and there have to be two types of experiments. One is a controlled experiment, in which XR is applied to language learning in the experimental group, but not in the control group. The other is setting a single group, which means that the same group will be given a pre-test and then a post-test after using XR for language learning
Data	The data provided in the article are enough to calculate the effect sizes, such as the sample size (N), Mean, standard deviation (SD), t-value, value of p, etc.
Access method	The article can be accessed *via* the internet

**Figure 1:**
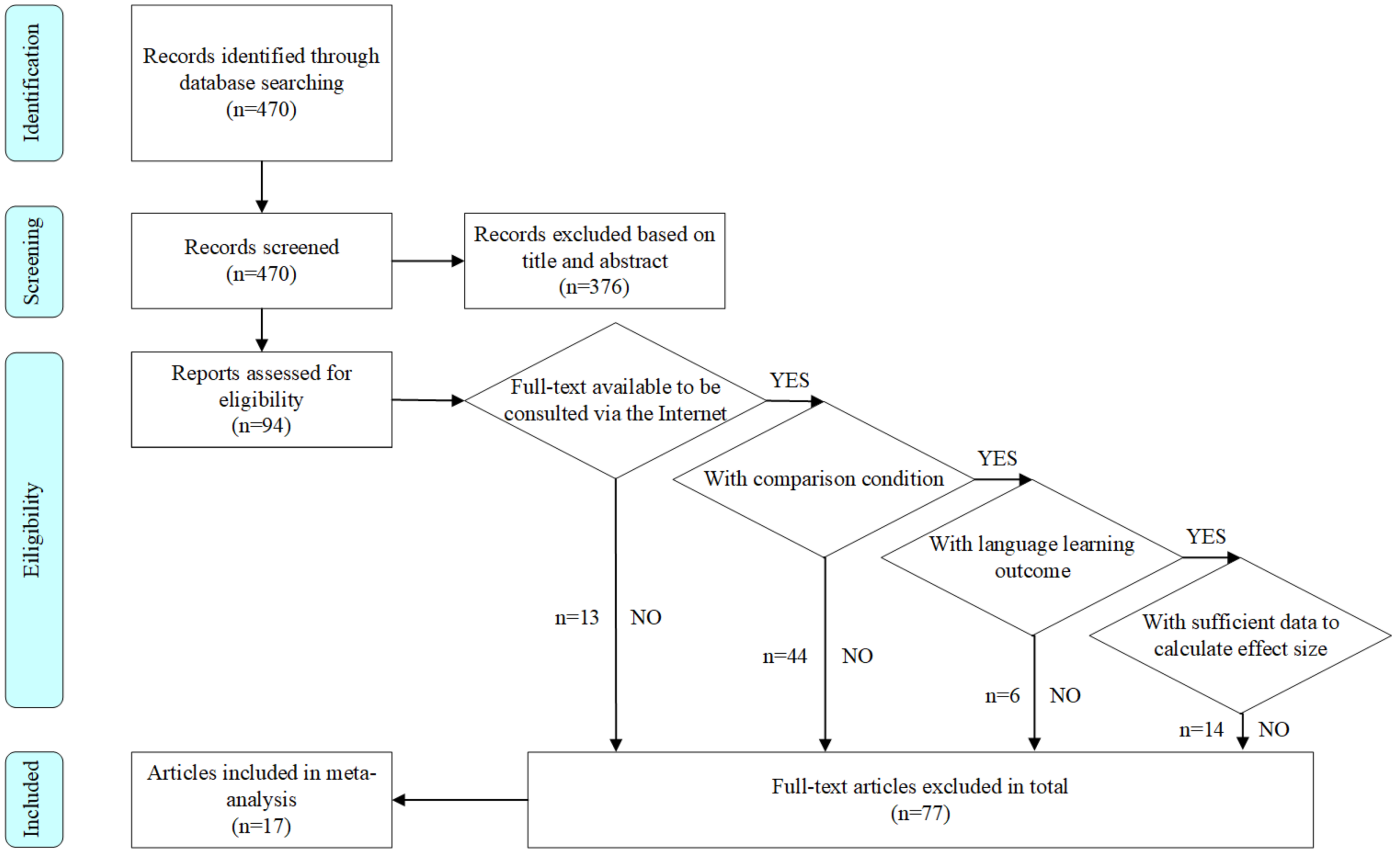
Flowchart for the article screening process.

When implementing the inclusion criteria, the present study referred to the execution process of Luo et al. (2021a), and combined it with the process of PRISMA to complete this step. First, three research team members examined the titles and abstracts of articles independently, and excluded 376 articles that were irrelevant to the research topic. When there was any disagreement during this stage of the process, the three researchers would discuss it and vote. Then, we carefully read the remaining articles and discarded 77 articles that did not fulfill the inclusion criteria in terms of the research methods used and data provided. This process was jointly completed by three research team members to ensure the reliability of the data screening process. In the above two rounds of screening, the consistency of the three team members was higher than 95%. After completing the above screening process, 17 articles were included in the meta-analysis (Figure 1).

After selecting the articles, the quality of the published research needed to be carefully evaluated. Since the 17 articles were published in academic journals or conference collections, they had undergone rigorous double-blind peer review, which ensured the quality of the research. To further ensure the quality of the study, we invited three professors who are experts in meta-analysis to review the 17 articles, mainly focusing on the rationality of the experimental design and the adequacy of data reporting. Finally, all three professors agreed on the quality of the studies, indicating that they met the requirements of a meta-analysis.

### Data coding and descriptive statistics

To facilitate statistical analysis and the calculation of effect size, we needed to code the features of the literature. The present study takes the following features into account:

- Characteristics of the article (title, year of publication)
- Population characteristics (age, educational level)
- Experimental design (number of subjects, technology type, target language, language skill)
- Experimental results (mean, standard deviation, value of *p*).

The above features were independently extracted and coded by two authors. According to the coding standard proposed by Lipsey and Wilson (2001), the coding of a meta-analysis should take independent samples as the coding unit; that is, if there are multiple independent samples in a study, we code them separately and yield multiple independent effect sizes. Next, the 17 articles consisting of 21 sets of independent samples were studied. Any problem encountered was solved by discussion and voting by the three team members. The consistency of coding was higher than 98%, indicating that the coding results had good reliability and validity.

After the coding, the research team found that the final 17 articles, with a total of 993 subjects included in the meta-analysis, were mainly concentrated in terms of publication year: they were mostly published after 2010, especially in the most recent 5 years (2017–2021). As for the type of XR used, VR was applied in 11 studies, and AR was examined in six studies. Studies before 2016 all used VR, and since then, AR was gradually adopted in the research. The educational level of subjects covered a wide range, from kindergarten to university. Among them, subjects in primary schools and universities were most frequently studied by researchers, appearing in seven and six studies, respectively. There were 13 studies that selected English as the target learning language, which may be explained by the fact that English is the most widely used language in the world.

## Results and discussion

After the preliminary literature reorganization and data coding work, the CMA software was used to perform detailed analyses in sequence: the effect value calculation, heterogeneity test, sensitivity analysis, publication bias analysis, and subgroup analysis.

### Effect size calculation

Effect size is an important indicator that estimates the magnitude of the effect or association between two or more variables (Snyder and Lawson, 1993). We used Hedges’ *g* (1981) because, compared with the method of calculating effect size brought up by Cohen (1969) and Glass (1976), this method is more suitable for small sample sizes. Based on Cohen’s *d* (1969), the effect sizes of 0.2, 0.5, and 0.8, respectively, corresponded to a small effect, medium effect, and large effect.

### Heterogeneity test

**Figure 2:**
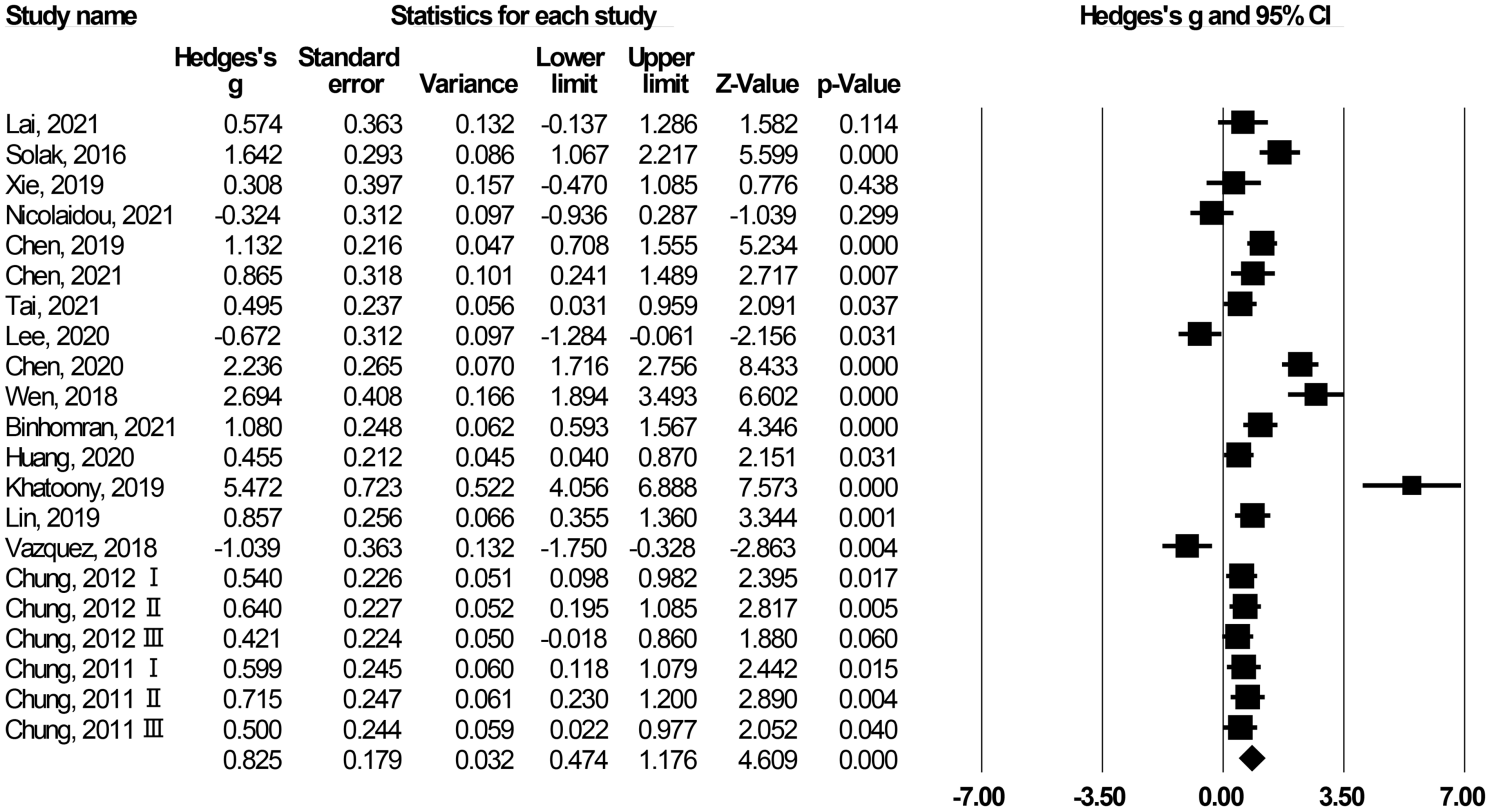
Forest plot for the impacts of XR on language learning.

As the experimental designs and characteristics of research subjects differed across the included articles, the findings on VR’s impact on language learning would be affected, thus there was likely to be heterogeneity in the measured effect sizes. A heterogeneity test was conducted to determine whether to perform a moderator analysis to examine the source of heterogeneity, on the one hand, and to decide whether to adopt a fixed-effects model or a random-effects model, on the other hand. Heterogeneity was tested using *Q*-tests. The results showed that *Q* = 177.356 (*p* < 0.001) and *I^2^* = 88.723%; the latter indicated that 88.723% of heterogeneity came from the differences in effect sizes. According to the standard set by Higgins et al. (2003), when *I^2^* is greater than 75%, this suggests a high degree of heterogeneity. Therefore, we adopted a random-effects model, and the summary effect size obtained was 0.825 (*p* < 0.001), indicating a large effect (Cohen, 1969). Figure 2 presents the forest plot of the study. The results suggest that the application of XR has a considerable effect on language learning. Figure 2 also shows that the 95% confidence interval is far higher than zero, which suggests that a large effect resulted from the message reflected in the research data itself, but not from random error.

Based on the above analyses, we can conclude that XR can significantly boost the learning effect, a finding that is consistent with the conclusions of much comprehensive research (Solak and Erdem, 2015; Qiu et al., 2021). Many scholars gave reasonable explanations for this. For example, as XR assumes the characteristics of deep immersive interaction and high involvement in learning, it is able to break the limitations of the traditional media applied in education to provide language learners with a realistic simulated language learning environment, and to effectively support their language learning (Pinho et al., 2008; Nicolaidou et al., 2021).

### Sensitivity analysis

**Figure 3:**
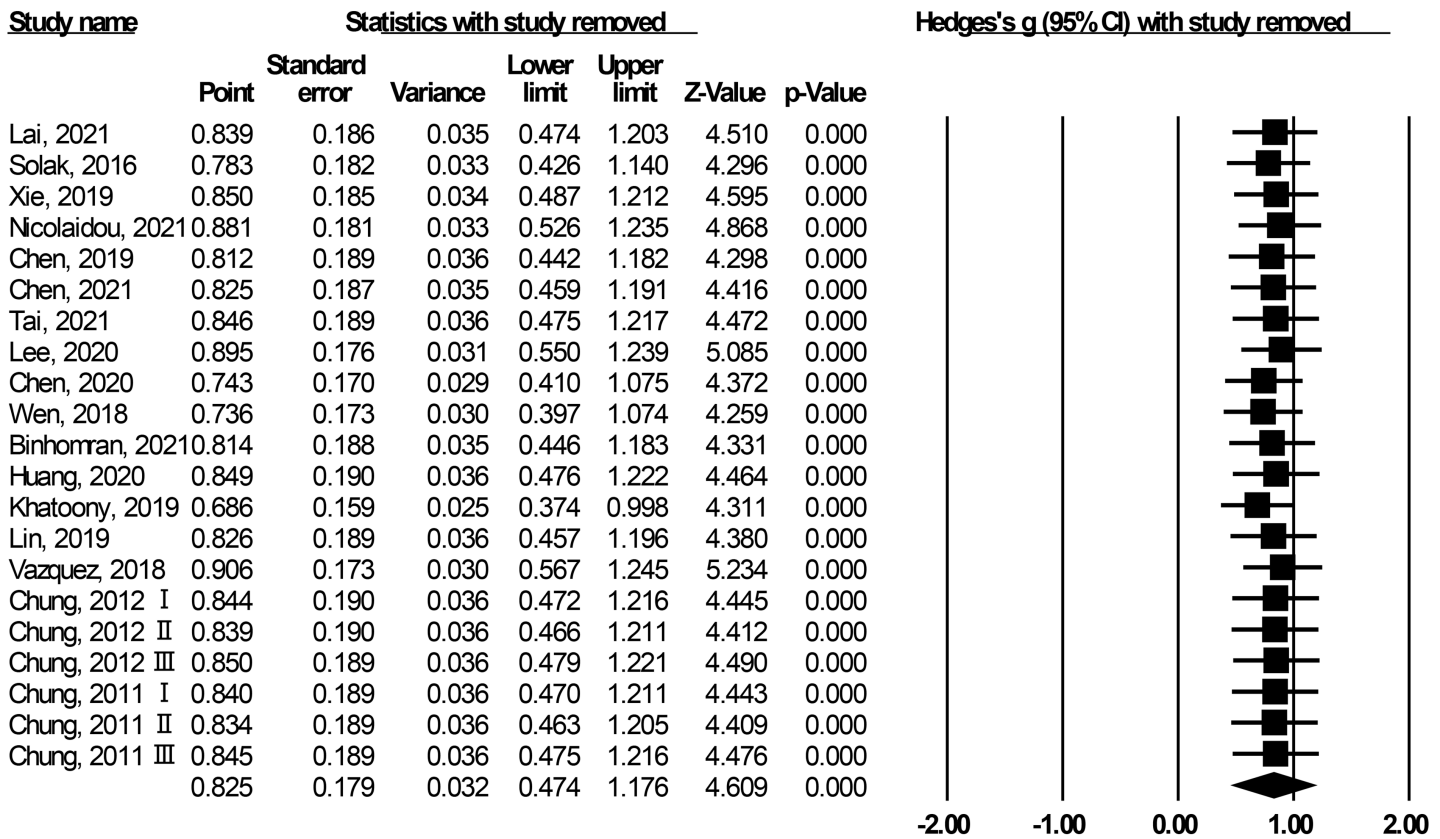
Results of the sensitivity analysis.

Sensitivity analysis can help the meta-analysis practitioner assess the confidence of their results (Elvik, 2005). The sensitivity analysis was performed using the One Study Removed method in CMA 3.0. After removing each included study, one by one, the effect size was merged to observe whether it deviated. The results are shown in Figure 3. No matter which study was excluded, the offset of the effect size was relatively stable [in the interval of (0.686, 0.906)], which ensured the robustness and reliability of the meta-analysis.

### Publication bias analysis

**Figure 4:**
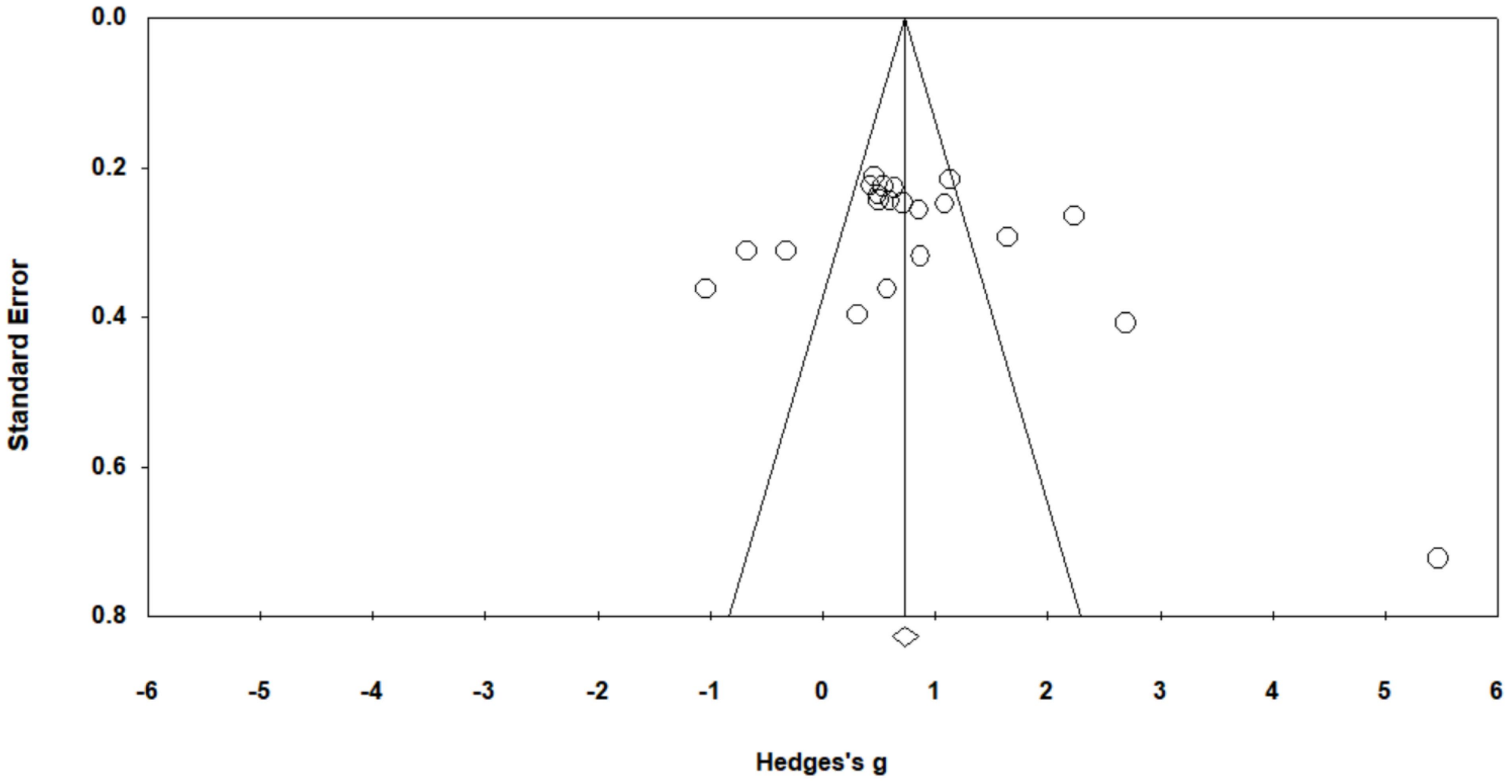
Funnel plot with effect sizes.

Amid a systematic review, the presence of publication bias means that the published articles cannot fully represent the overall study results in a field (Rothstein et al., 2006). To examine publication bias in this study, we used the funnel plot and the fail-safe *N*. As shown in Figure 4, the effect sizes are evenly distributed on the two sides of the aggregating effect size, preliminarily indicating that there is no serious publication bias in the selected studies. Because the funnel plot is only an initial and subjective test, the fail-safe *N* is needed to perform a further analysis. Rosenthal (1991) proposed that if *N* > 5 k + 10, publication bias is not likely to affect the results of a meta-analysis. The *N* of this study is 882, which is much larger than 115 (5 × 21 + 10), and is within the average range. Based on the analysis above, there is no significant publication bias in this study, which means that the results of the meta-analysis are robust and reliable.

### Subgroup analysis

Due to the high degree of heterogeneity in the samples, there is likely to be significant moderator variables. Thus, a subgroup analysis was conducted to determine the source of the heterogeneity, and to study the moderation effects of the sample characteristics (i.e., educational level, target language, technology type, language skill) on effect size.

#### Educational level

As shown in Table 2, according to the *Q*-statistics, the educational level of learners and XR does not have a significant impact on language learning achievement (*Q* = 4.096, *p* = 0.129). Apart from that, it is clear that, supported by XR, primary school learners achieved strong learning effects (*g* = 1.131, *p* < 0.001), as did middle school learners (*g* = 0.646, *p* < 0.001). When it comes to college learners, however, XR did not emerge as a significant booster of learning (*g* = 0.238, *p* = 0.658).

**Table 2:** Impacts of XR on the learning effects, by education level.

Educational level	*N*	*g*	*SE*	95% *CI*		*Z*	*p*	Between-group effects
				Lower	Upper			
Primary education	11	1.131	0.229	0.682	1.579	4.939	< 0.001	*Q* = 4.096*, p* = 0.129
Secondary education	3	0.646	0.157	0.338	0.953	4.115	< 0.001	
Higher education	6	0.238	0.537	−0.814	1.290	0.443	0.658	

*N*, number of samples; *g*, Hedges’ g (effect sizes); *SE*, standard error; *CI*, confidence interval.

To figure out the reason why college learners were not significantly influenced, we scrutinized a number of studies of this population. The analysis found that many learners were quite interested in XR, which received more attention than the learning material itself (Vázquez et al., 2018). In experimental studies, this phenomenon is similar to the Hawthorne effect. Vázquez et al. (2018) confirmed the above analysis: after a week spent familiarizing themselves with the XR device, the participants again conducted the experiment, with the experimental group using the VR; their learning effect began to grow significantly higher than that of the control group, who used the traditional learning method. Such a conclusion suggests that the cognitive cost, which resulted from learners’ unfamiliarity with the VR interface, can possibly account for the poor learning effect at the start. Similar explanations are mentioned in another study (Cheng et al., 2017). Meanwhile, Vázquez et al. (2018) also found that college freshmen were more accustomed to the traditional mode of learning (because they are used to using traditional education approaches in K–12 schooling), so in the face of the new technology being used as a new teaching mode, these individuals may be unable to adapt to the new technology, resulting in a poor learning effect.

Lee (2020) provide a different explanation. Their team discovered that merely applying emerging technologies would not necessarily promote students’ engagement and academic performance, and that innovative instructional design and new teaching principles were the right paths to foster language learning development. Their work offers a new perspective on technology-based study of language learning, reminds us to adopt a rational and objective attitude toward technology, and calls for a reexamination of language teaching that is integrated with technologies today (Luo et al., 2021a).

Xie et al. (2021) also analyzed and explained this problem. They argued that the plasticity of learners’ language learning ability would be greatly reduced after they become adults, especially in terms of pronunciation and fluency, and it was difficult for adult college students to make significant progress in language learning in a short period of time with the intervention of XR technology. Solak and Cakır (2016) confirmed this, suggesting that the application of AR technology can significantly improve the satisfaction and enthusiasm of young learners. Another explanation mentioned by Xie et al. (2021) was that learners in primary school and middle school had higher self-consciousness compared with college learners; in some studies that lasted for a long time, learners in primary and middle school would continue to learn after class, which swayed the results to indicate that learners who did not use the XR technology were more likely to have significant differences than those who used the XR technology in primary and middle school.

In fact, as explained by Vázquez et al. (2018) and Lee (2020), we can improve the experimental design to obtain some more stable and practically instructive studies. To eliminate participants’ inattention due to technical novelty, the learners can be exposed to the new technology environment for a period of time, helping them adapt to the equipment and the intervention learning mode (Gavgani et al., 2018). On this basis, we can eliminate some unnecessary interference factors, or we can use the Latin square design to exclude the above interference factors to reduce the influence of endogenous factors generated by the research design (Richardson, 2018). Undoubtedly, the research on this issue needs to be supplemented by more empirical studies.

#### Target language

It can be seen from the *Q*-statistics that there is a notable influence of the moderating effect of different target languages on the learning effect (*Q* = 16.128, *p* < 0.001; Table 3). When the target language is English, the application of XR could considerably promote the learning effect (*g* = 0.904, *p* < 0.001). There is no significant result for the learning of Chinese or for two other languages (Italian and Spanish) (*g* = 1.499, *p* = 0.209 and *g* = −0.657, *p* = 0.065, respectively).

**Table 3:** Impact of XR on the learning effects, by target language.

Target language	*N*	*g*	*SE*	95% *CI*		*Z*	*p*	Between-group effects
				Lower	Upper			
Chinese	2	1.499	1.193	−0.839	3.837	1.256	0.209	*Q* = 16.128, *p* < 0.001
English	17	0.904	0.172	0.567	1.241	5.262	< 0.001	
Other languages	2	−0.657	0.356	−1.356	0.041	−1.845	0.065	

*N*, number of samples; *g*, Hedges’ g (effect sizes); *SE*, standard error; *CI*, confidence interval.

XR can effectively promote English language learning, a conclusion that was supported by many studies (Khatoony, 2019; Chen and Hwang, 2020; Lee, 2020; Tai and Chen, 2021). The technical forms supporting English language learning were also very plentiful. In addition, the research on XR in English language learning started earlier and lasted a long time. In the long accumulation and practice, many teaching models and methods suitable for English teaching were formed, which undoubtedly improved the effect of English language learning after the technology intervention.

When reviewing studies of Chinese and Italian learners, we discovered that the research had a more detailed classification of language learning compared to work on English learners. For example, Xie et al. (2021) divided the language learning effect into five dimensions: content, fluency, vocabulary, pronunciation, and grammar. The results turned out to be vastly different. On content and vocabulary, the experimental group that used VR technology obtained notable greater learning performance than the control group, but there was no marked significance between the two groups in terms of fluency, pronunciation, or grammar. After synthesizing various studies using a meta-analysis, we found that VR does not exert a great impact on learning in general. Still, we cannot arbitrarily assume that XR will not play a prominent role in the learning of Chinese or Italian in all respects. It is important that we carefully consider the question: On which language skills does XR have an impact? Further discussion and analysis of this is provided in section “language skill”.

In fact, as all the included studies target the learning of a single language, it is hard to tell the difference in learning effects between languages and how different languages impact technology-integrated learning from the conclusions of these studies. However, we suppose that the features of diverse languages would, to some degree, moderate the impact of XR on language learning; in addition, the differences in the learning effects of different target languages may also come from the maturity of XR in the language learning field. More empirical research should analyze and explain the reason and mechanism behind this observation.

#### Technology type

The samples of this study contained VR and AR technologies, which both significantly contribute to language learning outcomes (Table 4). Yet, it is noteworthy that they differ in terms of the degree of their impact on learning. VR has a huge impact on language learning (*g* = 1.101, *p* = 0.001), while AR’s impact is above average (*g* = 0.709, *p* < 0.01). Meanwhile, the different technologies do not show a significant difference in the moderating effect of language learning (*Q* = 0.878, *p* = 0.349).

**Table 4:** Impacts of XR on the learning effects, by technology type.

Technology type	*N*	*g*	*SE*	95% *CI*		*Z*	*p*	Between-group effects
				Lower	Upper			
VR	15	0.709	0.205	0.308	1.111	3.462	0.001	*Q* = 0.878, *p* = 0.349
AR	6	1.101	0.387	0.567	1.814	3.024	< 0.01	

*N*, number of samples; *g*, Hedges’ g (effect sizes); *SE*, standard error; *CI*, confidence interval.

Based on analyses of several typical studies (Solak and Cakır, 2016; Binhomran and Altalhab, 2021; Lai and Chen, 2021), we may assume that the difference in the impact on learning between VR and AR, to a large extent, originates from the characteristics of the technology itself. Compared with VR, AR emphasizes a real sense of presence and is therefore believed to help facilitate learning and visualization of abstract concepts in the mind (Gün, 2014). But it is a pity that only one XR technology was applied in all included studies, and there is a dearth of research on the difference between VR and AR, which makes it impossible for us to accurately determine the exact characteristic of the technology that leads to the different impacts on learning. The answer to this problem awaits further explanation from future empirical research.

Although the analysis in this present study cannot evaluate the differences between VR and AR when applied to language learning, some common factors of the two can be analyzed. Lee (2020) not only proved that XR would have better effects on language learning through experimental research, but also further analyzed the internal mechanism through interviews. In this setting, a student said that XR made him interested in exploratory learning, while in the traditional learning mode, information can only be learned and obtained by reading. In other words, the realistic situations provided by XR were the key for learners to achieve better learning outcomes. Of course, some studies found that the advantages of XR also consisted of improving students’ understanding and enthusiasm for learning (Binhomran and Altalhab, 2021). In addition, previous studies have demonstrated that XR techniques can significantly improve memory retention time compared with traditional research methods (Pérez-López and Contero, 2013). As language learning was a learning activity requiring memory, this feature of XR matched the properties of language learning activities, which gave the application of XR in language learning extraordinary advantages and unique value.

Finally, it is worth noting that any technology intervention requires an adaptation period for the learners, especially for some complex technologies, and some even require corresponding training before use (Binhomran and Altalhab, 2021). To our relief, however, almost all articles included in the present research have considered this in the design of their experiments. In addition, teachers must pay attention to this point so that students’ knowledge and skills can benefit from the new technology and some of the adverse effects caused by technical barriers can be reduced.

#### Language skill

Language skills were classified into five categories (vocabulary, speaking, grammar, reading, listening) according to the descriptions in the articles. The *Q*-statistics revealed no significant differences in the moderating effects of different language skills on language learning (*Q* = 5.346, *p* = 0.375; Table 5). Moreover, the learning of the five language skills is noticeably influenced by XR. Among them, speaking is most significantly promoted (*g* = 2.100, *p* < 0.01), and vocabulary shows a high degree of impact (*g* = 0.762, *p* < 0.01). The remaining three skills (grammar, listening, reading) are moderately impacted (*g* = 0.590, *p* < 0.001; *g* = 0.495, *p* < 0.05; *g* = 0.457, *p* < 0.01). The influence of XR on the five language skills occurred from the largest to the smallest as follows: speaking, vocabulary, grammar, listening, and reading.

**Table 5:** Impacts of XR on the learning effects, by language skill.

Language skill	*N*	*g*	*SE*	95% *CI*		*Z*	*p*	Between-group effects
				Lower	Upper			
Vocabulary	9	0.762	0.287	0.199	1.325	2.652	< 0.01	*Q* = 5.346, *p* = 0.375
Speaking	4	2.100	0.771	0.588	3.612	2.723	< 0.01	
Reading	2	0.457	0.165	0.134	0.780	2.773	< 0.01	
Listening	1	0.495	0.237	0.031	0.959	2.091	< 0.05	
Grammar	3	0.590	0.131	0.333	0.847	4.495	< 0.001	

*N*, number of samples; *g*, Hedges’ g (effect sizes); *SE*, standard error; *CI*, confidence interval.

Speaking has been noted by a number of studies (Khatoony, 2019; Chen et al., 2021; Xie et al., 2021) to have been aided by the integration of XR into language learning. This phenomenon is not difficult to understand given the fact that XR is famous for its authenticity and immersion, and that the realistic experience provides an excellent practice environment for speaking. Vocabulary is also greatly enhanced by XR technology, as expected; Pérez-López and Contero (2013) demonstrated that the intervention of XR can significantly improve learners’ memory retention ability, so it is undoubtedly a great benefit to vocabulary memorization skills.

To learn more about the differences in the learning effects of diverse language skills, Xie et al. (2021) carried out systematic qualitative research based on experimental studies, and gave insightful explanations of their conclusions in the form of interviews. Quantitative research informs the relationship between research variables, while qualitative research provides insights into the causal logic behind relationships *via* case-by-case in-deep analysis. Through the interviews, Xie et al. (2021) found that, for some language skills, such as grammar and reading, it is difficult to make considerable improvement in a short time when a certain stage is reached; moreover, a large amount of input from a native speaker is needed to enhance these skills. Vocabulary, by contrast, is a kind of language skill that can be enhanced rapidly with the help of technology. This explains why the learning effects of grammar, listening, and reading were lower than that of speaking and vocabulary. The above conclusion is also supported by other studies (e.g., Bongaerts et al., 1997).

Examining the differences in language skills after technology intervention can provide learners and teachers with occasions to apply extended realistic technology and to avoid some adverse effects caused by the abuse of technology (such as blindly pursuing technology, but not paying attention to learning methods and teaching methods). Many scholars have pointed out that the greatest value of XR for language learning is to provide immersive realistic situations so that learners can conduct language learning as if they were in a foreign language community (Chung, 2011, 2012; Xie et al., 2021). XR is of great value in improving speaking, but this study also reminds us that we should not rely too much on XR in the practice of reading and other skills. We should also maintain a rational attitude toward emerging technologies, and actively explore alternative models that are conducive to improving learners’ reading and other skills (Luo et al., 2021a).

## Conclusion and prospects for future research

### Conclusion and implications

The present study uses a systematic meta-analysis to review the application of XR in language learning over the past 20 years, reveals the learning effects of technology intervention in language learning, and answers the three questions raised in the introduction. First, through the comprehensive calculation of the effect size of the included articles, XR is confirmed to have a large positive effect on learning (effect size = 0.856, *p* < 0.001). Second, adjusting the analysis of the variables, such as education level, this study finds that the target language is an important adjustment variable affecting the results of the study, and different techniques have different benefits for language learning. This demonstrates that different languages and technologies have different features, and we need to select technologies according to the features of the language itself to make them match. Some researchers also point out that technology and its derivative products need to be designed according to language characteristics (Xie et al., 2021). This suggestion is undoubtedly of great significance. Finally, the present study also points out some meaningful research concerns, including:

1. How does the difference between VR and AR influence the learning effect of language learning, which helps us to choose which technology to use? There is a lack of comparative study of VR and AR in the existing research on language learning.
2. The relationship between the language learning effects presented by different language types and the characteristics of the language itself needs to be further analyzed. Existing studies are all focused on a single language, which is valuable but insufficient. If necessary, the mother language can also be included as an influencing factor.
3. The relationship between the differences in language learning effects and the characteristics of education levels has not been explored. Existing studies only examine a single learning stage, and further empirical studies are needed.

The present study highlights some practical implications for the use of XR in language learning. First, based on the discussion of the results, blind application of these emerging technologies may not necessarily bring about the desired results (Xie et al., 2021). We need to understand how these technologies improve learners’ performance after they are involved in language learning, and mastering these mechanisms can help these technologies to play a better auxiliary role. For example, XR has remarkable advantages in improving speaking, which is closely related to the sense of presence and immersion provided by XR (Radianti et al., 2020). Second, educators should evaluate the maturity of relevant technologies in the application field and take it as a condition for whether to apply relevant technologies. In the subgroup analysis of language types, experimental studies with English as the target language often have better learning effects, although there are factors of language characteristics, it is also closely related to whether the technology is mature in terms of product design in other language teaching fields. The bold application of immature technology in language learning may bring adverse effects. In addition, some scholars have emphasized some principles of the use of emerging technologies in empirical studies, pointing out that what really matters is not the use of these technologies, but the use of innovative teaching principles in language teaching (Lee, 2020), which is of great practical significance for front-line teaching staff. It is necessary for educators to examine the existing technology rationally and objectively and to introduce it into teaching activities scientifically and reasonably. Finally, XR technologies still face some user experience issues (Luo et al., 2021a), such as physical discomfort of learners, security risks, and low technical stability. These factors need to be considered when the application of XR is extended.

### Limitations and prospects for future research

Although the effects of systematic error and random error on the conclusion have been accounted for as much as possible, there are still some design flaws and other factors that affect the reliability of this research. First, the present study only takes learning outcomes into consideration, without paying attention to the emotions, attitudes, and psychological state of learners. In addition, as the included studies did not explore the development of higher-order thinking in language acquisition, the present study did not conduct a detailed analysis of this topic.

Second, as analyzed in the present study, the design of the experiment may affect its conclusions, and the nature of the experimental design (i.e., that it is conducted over a short period of time) may influence the learning effects because students are not very familiar with the XR equipment. Among the included literature, some studies were long experimental studies, lasting up to 5 or 6 weeks (e.g., Chen and Hwang, 2020; Binhomran and Altalhab, 2021), and some were as short as 1–2 h (e.g., Huang et al., 2020). Future work might account for this difference.

Third, during the study, a series of reliability tests, which include the heterogeneity test, sensitivity analysis, and publication bias analysis, were undertaken, and the effect of publication bias has been proven non-significant on a technical level. Even so, since the study focused on published literature, if the primary researcher gave up publishing a paper due to the non-significance of the findings, or an editor thought that it was meaningless to publish an insignificant study, publication bias as such would noticeably impact the reliability of a meta-analysis’s conclusions (Coursol and Wagner, 1986).

In spite of the above unavoidable problems, we have reason to believe that the integration of XR into language learning will continue; thus, related studies require constant evaluation and updates, which has also been recognized by many researchers (Chen and Hwang, 2020; Luo et al., 2021a,b). The limitations pointed out above need to be addressed in the future. For example, we can expand the data source, perhaps by including ERIC and other highly related databases, or we can include in the search databases that are highly relevant to pedagogy (e.g., ERIC). By doing so, empirical research on more languages could be accessed to make the subgroup analysis of language types more reliable. In terms of publication bias, over recent years this kind of cognitive bias has been reduced dramatically with the efforts of some scholars who have conducted meta-analyses (Halpern et al., 2005). Therefore, with the improvement of methodology and research design, scholars will be able to reach more accurate conclusions on this topic.

## Data availability statement

The raw data supporting the conclusions of this article will be made available by the authors, without undue reservation.

## Author contributions

All authors contributed to the conception of the idea, screening the literature, analyzing the data, and writing the manuscript. All authors contributed to the article and approved the submitted version.

## Funding

This research was supported by the General Scientific Research Projects of Zhejiang Provincial Department of Education (grant no. GZ20491080003), Education Science Planning Project of Zhejiang Province (grant no. 2021SCG236), Philosophy and Social Science Planning Project Fund Project of Zhejiang Province (grant no. 17GXSZ19YB), Teaching Reform Project of Zhejiang University of Technology (grant no. JG2022064), and National University Student Innovation and Entrepreneurship Training National Project of China (grant nos. 202210337027 and 202210337003).

## Conflict of interest

The authors declare that the research was conducted in the absence of any commercial or financial relationships that could be construed as a potential conflict of interest.

## Publisher’s note

All claims expressed in this article are solely those of the authors and do not necessarily represent those of their affiliated organizations, or those of the publisher, the editors and the reviewers. Any product that may be evaluated in this article, or claim that may be made by its manufacturer, is not guaranteed or endorsed by the publisher.
